# Matching synaptic type with postsynaptic firing class shapes the encoding of either stimulus rate or rate change

**DOI:** 10.1186/1471-2202-12-S1-P321

**Published:** 2011-07-18

**Authors:** Ashutosh Mohan, Mark D McDonnell, Christian Stricker

**Affiliations:** 1 John Curtin School of Medical Research, Australian National University, Canberra, ACT 2601, Australia; 2 Institute for Telecommunication Research, University of South Australia, Mawson Lakes, SA 5095, Australia; 3 ANU Medical School, Australian National University, Canberra, ACT 2601, Australia

## 

The synergy between synaptic and postsynaptic firing dynamics in shaping neuronal encoding has not been explored. We show that by matching short-term synaptic dynamics with postsynaptic firing class, either stimulus rate or a rate change are encoded. The result may be relevant to understand the function of cortical microcircuits.

Short-term synaptic dynamics and firing dynamics of neurons can each be classified into two types. In short-term plasticity, type 1 synapses show release-dependent depression and constant rate of recovery. They appear to encode a stimulus rate change. Type 2 synapses show release-independent depression and faster recovery at higher stimulus frequencies. They can follow the stimulus rate [1]. Postsynaptic firing characteristics conform either to class 1 or 2 on the basis of phase-reset curves (PRCs) [2]. Class 1 neurons can fire at arbitrarily low frequencies and do not exhibit spike frequency adaptation. Class 2 neurons exhibit subthreshold oscillations and spike frequency adaption.

We investigate if different combinations of synaptic and postsynaptic dynamics can shape neuronal encoding.

**Figure 1:**
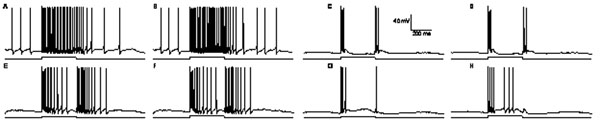
Spiking response when a class 1 neuron is matched with type 2 synaptic inputs (A,B) upon altering stimulus rate (below). Class 2 neuron matched with type 1 synaptic inputs (C,D). Class 1 neuron receiving type 1 synaptic inputs (unmatched; E,F). Class 2 neuron receiving type 2 synaptic inputs (unmatched; G,H). Average synaptic stimulus frequency altered for 400 ms from 10 to 30 Hz (A,C,E,G) and 10 to 40 Hz (B,D,F,H).

Using NEURON, we simulated biophysically realistic cells with class 1 or 2 firing dynamics [3] that receive 2000 unsynchronized type 1 or 2 synaptic inputs [1]. When class 1 neurons are matched with type 2 synaptic inputs the average synaptic stimulus rate is encoded in the firing rate (Fig. 1A,1B). When class 2 neurons and type 1 inputs are matched, the rate change but not its magnitude is encoded (Fig. 1C ,1D). For unmatched cases, rate and rate change are encoded to different extents (Fig. 1E-1H), suggesting both synaptic and postsynaptic dynamics shape encoding.

Spiny stellate cells in the layer IV microcircuit preferentially receive inputs from type 1 synapses while star pyramidal neurons receive type 2 inputs [4]. Even though the PRC for these cell types have not been characterized yet, our results pinpoint the possibility that in layer IV rate changes are encoded in the network of spiny stellate and stimulus rate in that of star pyramidal cells.
